# Association of Circulating ANGPTL8 Levels With Renal Dysfunction: A Case-Control Study

**DOI:** 10.3389/fpubh.2021.710504

**Published:** 2021-09-07

**Authors:** Xiaoyu Meng, Huajie Zou, Danpei Li, Peng Yu, Li Huang, Jianhua Zhang, Wenjun Li, Xuefeng Yu

**Affiliations:** ^1^Division of Endocrinology, Department of Internal Medicine, Tongji Hospital, Tongji Medical College, Huazhong University of Science and Technology, Wuhan, China; ^2^Branch of National Clinical Research Center for Metabolic Diseases, Wuhan, China; ^3^Computer Center, Tongji Hospital, Tongji Medical College, Huazhong University of Science and Technology, Wuhan, China

**Keywords:** ANGPTL8, eGFR, renal dysfunction, creatinine, chronic kidney disease, case-control study

## Abstract

**Background:** Chronic kidney disease (CKD) is recognized as a major public health problem with high morbidity and mortality worldwide. Recently, angiopoietin-like protein 8 (ANGPTL8) was found to regulate lipid metabolism. Previous studies suggested that serum ANGPTL8 levels increased in patients with diabetes, especially in diabetic patients with albuminuria. This study aimed to investigate the association between circulating levels of ANGPTL8 and kidney function in the general population.

**Methods:** The subjects were patients with renal dysfunction [estimated glomerular filtration rate (eGFR) <60/min/1.73 m^2^] from Risk Evaluation of cAncers in Chinese diabeTic Individuals: a lONgitudinal study (the REACTION study). Each case was matched by age, sex, and body mass index (BMI) with one control whose eGFR was ≥ 90 ml/min/1.73 m^2^. The case and control groups were compared using a paired *t*-test. Binary logistic regression analysis was used to calculate the odds ratio (OR) of renal dysfunction (RD).

**Results:** Among 135 case-control pairs, circulating ANGPTL8 levels were elevated in patients with RD compared to control subjects [799.96 (410.12-1086.44) vs. 609.58 (365.13-740.06) pg/ml, *p* < 0.05]. Partial correlations showed that ANGPTL8 levels were negatively correlated with eGFR (r = −0.26, *p* < 0.05). Multivariable-adjusted binary logistic regression analysis showed that elevated ANGPTL8 levels were associated with an increased risk of RD (OR in quartile 4 vs. 1, 3.80; 95% CI, 1.71-8.41). Interestingly, the association between ANGPTL8 levels and RD was consistent with the overall findings in both nondiabetic individuals (OR, 1.44; 95% CI, 1.09 to 1.91) and diabetic patients (OR, 2.71; 95% CI, 1.13-6.49) in the subgroup analyses. Furthermore, the estimates for this association were also significant in females (OR, 2.12; 95% CI, 1.33-3.37), individuals aged > 60 years (OR, 1.55; 95% CI, 1.16-2.07), individuals with a BMI <24 (OR, 1.66; 95% CI, 1.16-2.39), and individuals without hyperlipidaemia (OR, 1.61; 95% CI, 1.16-2.23) (all *p*-values <0.05).

**Conclusion:** Elevated circulating ANGPTL8 levels were associated with increased risk of RD in the general population, especially among females, individuals aged > 60 years, individuals with a BMI < 24, individuals without diabetes mellitus, individuals with diabetes mellitus (DM), and individuals without hyperlipidaemia. This finding implies that ANGPTL8 may play a role in the pathological process of RD.

## Introduction

Chronic kidney disease (CKD) is defined as kidney damage caused by a variety of factors, including diabetes, hypertension, autoimmune diseases, lipid dysregulation, and urinary tract infections (1, 2). CKD is recognized as a major public health problem with high morbidity and mortality worldwide, and it requires substantial financial and social resources (3–5). Recently, the global morbidity of CKD has been as high as 11-13% (6). According to the 2010 Global Burden of Disease study, CKD was ranked 27th in the list of causes of total global deaths in 1990 but rose to 18th in 2010, and the ranking of chronic kidney disease has also risen in recent years (7, 8). CKD is characterized by a decrease in renal function, as indicated by the estimated glomerular filtration rate (eGFR); unfortunately, there are no obvious clinical symptoms in the early stage until severe damage occurs. Early identification, diagnosis, and treatment are important to prevent disease progression because progressive CKD is associated with many adverse outcomes, including end-stage kidney disease, cardiovascular disease, and increased mortality (7, 8). There is a lack of sensitive and specific biomarkers to diagnosing and treating CKD and novel biomarkers urgently need to be identified.

Angiopoietin-like protein 8 (ANGPTL8), which is also called betatrophin, “refeeding induced in fat and liver” (RIFL), lipasin, TD26, and C19orf80, is an atypical member of the angiopoietin-like protein family. ANGPTL8 is secreted mainly from the liver in humans and from adipose tissue and the liver in mice (9–12). ANGPTL8 participates in the regulation of triglyceride (TG) metabolism by inhibiting the activity of lipoprotein lipase (LPL) (13).

Previous studies found that ANGPTL8 levels were increased in diabetic patients, especially in patients with diabetic nephropathy (14–17). Yang et al. found that circulating ANGPTL8 levels were significantly increased in type 2 diabetic patients with albuminuria and that ANGPTL8 was positively correlated with the urine albumin-to-creatinine ratio (ACR) in this population (17). A negative correlation between ANGPTL8 levels and the eGFR was also reported in a study by Maurer et al. (14). The present study aims to explore the association between ANGPTL8 levels and RD in the general population as well as diabetic patients.

## Subjects and Methods

### Study Participants

The participants in the present study were recruited from Hubei Province in China during 2011–2012 as part of Risk Evaluation of cAncer in Chinese diabeTic Individuals: a lONgitudinal study (the REACTION study), which was conducted among 259,657 adults aged 40 years and older in 25 communities across mainland China during 2011–2012 (baseline) and which invited participants to attend follow-up visits during 2014–2016 (18–20). At baseline, a comprehensive set of questionnaires, clinical measurements, oral glucose tolerance tests (OGTTs), and laboratory examinations were conducted following standardized protocols for all of the participants. RD was defined as an eGFR of <60 ml/min/per 1.73 m^2^ and/or the presence of markers of renal injury for more than one trimester (6). The eGFR was calculated based on Modification of Diet in Renal Disease for Chinese, as follows: eGFR (ml^·^min^−1·^1.73 m^−2^) = 175 × Serum creatinine^−1.234^ × Age^−0.179^ (×0.79, if female) (21). Each case was matched by age, sex, and body mass index (BMI) with one control whose eGFR was ≥90 ml/min/1.73 m^2^.

The Committee on Human Research at Tongji Hospital, Tongji Medical College, Huazhong University of Science and Technology, approved the study protocol, and all of the participants provided written informed consent. All of the methods were performed in accordance with the relevant guidelines and regulations.

### Clinical and Biochemical Evaluation

Data were collected from local community clinics at baseline and follow-up visits. As previously described in the REACTION study (20), information on sociodemographic characteristics, lifestyle factors, medical history, and family history were collected by trained staff using a standard questionnaire.

### Measurement of ANGPTL8 Levels

Blood samples were collected after overnight fasting. Serum was obtained after centrifugation, aliquoted, and then stored at −80°C. ANGPTL8 levels in the fasting serum were quantified using commercially available ELISA kits (Eiaab Science, Wuhan, China; Catalog No. E11644h) with an intra-assay coefficient of variation (CV) of ≤ 6.5% and an inter-assay CV of ≤ 9.2% (provided by the manufacturer). The procedures were performed according to the manufacturer's instructions. All of the samples were analyzed in duplicate.

### Statistical Analysis

The baseline characteristics of the participants are presented as medians (interquartile ranges, IQRs) for asymmetrically distributed data, as percentages and frequencies for continuous variables, and as numbers (proportions) for categorical variables. The highest and lowest 0.5% of data points for ANGPTL8 levels were trimmed. The data were tested for a normal distribution using the Kolmogorov-Smirnov test. We obtained *p*-values for differences between groups using a paired *t*-test or a non-parametric test for continuous variables that were normally distributed or abnormally distributed, respectively, and using the χ2 test for categorical variables. The correlations between variables were assessed using Spearman correlation analysis and partial correlation analyses by controlling for covariates, including age, sex, BMI, lipid profiles, and glucose profiles. Binary logistic regression analysis was conducted to calculate odds ratios (ORs) and 95% confidence intervals (CIs) for renal dysfunction in quartiles of ANGPTL8 levels. We adjusted multivariable models for age, sex, BMI, glycated hemoglobin A1c (HbA1c), high-density lipoproteins (HDL), low-density lipoproteins (LDL), TG, cholesterol, fasting plasma glucose (FPG), 2 h postprandial blood glucose (2hPG), alanine transaminase (ALT), aspartate aminotransferase (AST), DM and hyperlipidaemia. Subgroup analyses were conducted based on sex, age, BMI, history of DM, or hyperlipidaemia. A two-tailed *p*-value <0.05 was considered statistically significant. All statistical analyses were performed using SPSS software (version 26.0) and Stata software (version 11.0).

## Results

### Characteristics of the Study Population

The baseline characteristics of the study participants are shown in Table 1. Among 135 case-control pairs, no significant differences in age, sex, BMI, HbA1c, HDL, LDL, total cholesterol, TG, FPG, 2hPG, ALT, and AST data were detected between the case and control groups. The eGFR values in the case group were lower than those in the control group [45.91 (38.29-55.89) vs. 112.24 (101.13-123.45) ml/min/1.73 m^2^; *p* < 0.001], and creatinine levels were higher in the case group [142.81 (105.30-154.00) vs. 63.96 (56.20-69.30) μmol/L; *p* < 0.001].

**Table 1:** Clinical and biochemical parameters for non-renal dysfunction subjects and renal dysfunction patients.

**Characteristics**	**Non-renal dysfunction**	**Renal dysfunction**	**All**	***P*-value**
*N* (%)	135 (50)	135 (50)	270 (100)	
Age (years)	66.24 (61.00-74.00)	66.30 (60.00-74.00)	66.27 (61.00-74.00)	0.934
Male sex *n* (%)	58 (43)	58 (43)	116 (43)	1.000
BMI (kg/m^2^)	23.25 (20.88-24.90)	23.15 (20.60-25.14)	23.20 (20.79-25.07)	0.926
ANGPTL8 (pg/ml)	609.58 (365.13-740.06)	799.96 (410.12-1086.44)	704.77 (381.99-924.50)	0.002
eGFR (ml/min/1.73 m^2^)	112.24 (101.13-123.45)	45.91 (38.29-55.89)	79.08 (50.49-111.63)	0.000
Creatinine (μmol/L)	63.96 (56.20-69.30)	142.81 (105.30-154.00)	103.38 (61.28-120.28)	0.000
HbA1c (%)	5.96 (5.50-6.00)	6.00 (5.50-6.10)	5.98 (5.50-6.10)	0.319
HDL (mmol/L)	1.50 (1.23-1.71)	1.42 (1.16-1.59)	1.46 (1.19-1.67)	0.072
LDL (mmol/L)	2.83 (2.26-3.36)	2.91 (2.22-3.48)	2.87 (2.23-3.42)	0.987
Total cholesterol (mmol/L)	4.99 (4.38-5.49)	5.09 (4.25-5.72)	5.04 (4.26-5.57)	0.979
TG (mmol/L)	1.47 (0.84-1.63)	1.58 (0.99-1.88)	1.53 (0.92-1.74)	0.069
FPG (mmol/L)	5.66 (4.89-5.68)	5.97 (4.98-5.87)	5.82 (4.94-5.77)	0.121
2h PG (mmol/L)	7.54 (5.80-7.93)	7.93 (5.86-8.36)	7.73 (5.84-8.21)	0.450
ALT (U/L)	13.70 (9.00-17.00)	13.87 (8.00-18.00)	13.79 (9.00-17.25)	0.946
AST (U/L)	23.55 (18.00-26.00)	25.54 (19.00-27.00)	24.54 (18.00-26.00)	0.206

*BMI, body mass index; eGFR, estimated glomerular filtration rate; HbA1c, glycated haemoglobinA1c; HDL, high-density lipoproteins; LDL, low-density lipoproteins; TG, triglycerides; FPG, fasting plasma glucose; 2h PG, 2h postprandial blood glucose; ALT, alanine transaminase; AST, aspartate aminotransferase*.

### Association Between ANGPTL8 Levels and Renal Function

**Figure 1:**
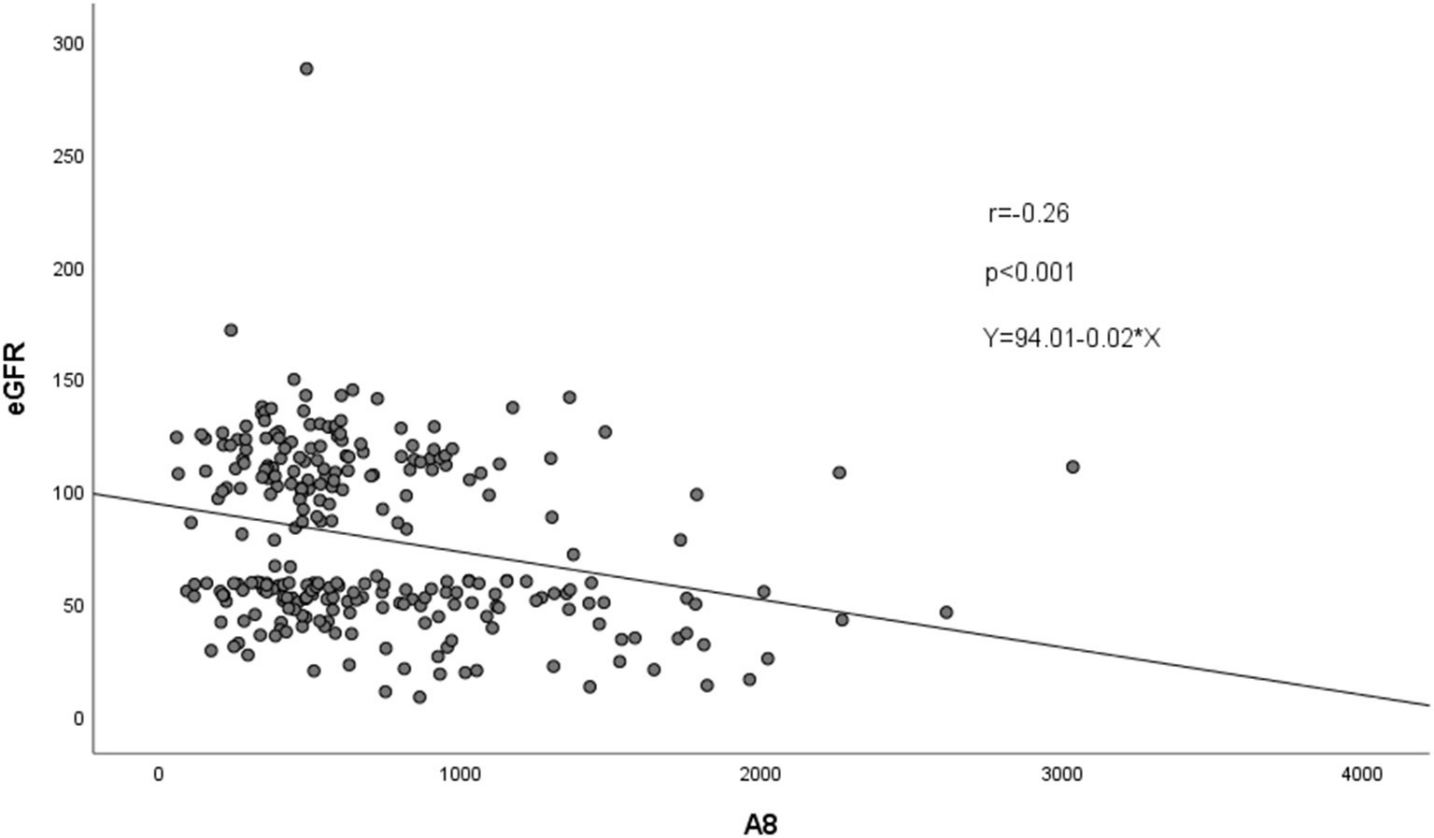
Scatter and linear plots of ANGPTL8 levels and renal function.

The circulating ANGPTL8 levels were significantly increased in the case group compared to the control group [799.96 (410.12-1086.44) vs. 609.58 (365.13-740.06) pg/ml, *p* < 0.05; Table 1]. Furthermore, the serum ANGPTL8 levels were inversely associated with the eGFR (r = −0.26, *p* < 0.05; Figure 1). Binary logistic regression analysis showed that compared with individuals in the first quartile of ANGPTL8, patients in the fourth quartile of ANGPTL8 levels had a 3.27-fold increased risk of RD (OR = 3.27, 95% CI = 1.60-6.68) in the unadjusted model. Notably, the relationship between ANGPTL8 levels and RD still existed after adjusting for covariables (OR = 3.80, 95% CI = 1.71-8.41; Table 2). After Z-transform standardization, the OR for RD was 1.68 (95% CI = 1.24-2.28) per unit increase in the Z score of ANGPTL8 levels.

**Table 2:** Risk ratios for clinical outcomes according to quartiles of ANGPTL8.

**Outcome**		**Model1**	**Model2**	**Model3**
RD	Q1 (Reference)	1	1	1
	Q2 (OR, 95% CI)	1.10 (0.56-2.17)	1.11 (0.55-2.23)	0.99 (0.47-2.07)
	Q3 (OR, 95% CI)	1.10 (0.56-2.17)	1.19 (0.59-2.38)	1.22 (0.59-2.52)
	Q4 (OR, 95% CI)	3.27 (1.60-6.68)	3.59 (1.69-7.61)	3.80 (1.71-8.41)
	Per unit increase in Z score	1.55 (1.18-2.03)	1.59 (1.20-2.11)	1.68 (1.24-2.28)

*Model 1 was unadjusted*.

*Model 2 was adjusted for age, sex and BMI*.

*Model 3 was adjusted for all variables in model 2 plus HbA1c, HDL, LDL, TG, cholesterol, FPG, 2h PG, ALT, AST, DM, and hyperlipidaemia*.

### Association Between ANGPTL8 Levels and Metabolic Variables

The correlations between the ANGPTL8 levels and metabolic variables in all participants were analyzed by Spearman correlation analysis. As shown in Table 3, after controlling for multiple variables, ANGPTL8 levels were positively correlated with age (r = 0.20, *p* < 0.05), BMI (r = 0.15, *p* < 0.05), creatinine (r = 0.30, *p* < 0.001), and AST (r = 0.25, *p* < 0.001) but negatively correlated with the eGFR (r = −0.27, *p* < 0.001) and LDL (r = −0.13, *p* < 0.05). There were no associations between ANGPTL8 levels and HbA1c, HDL, total cholesterol, TG, FPG, or ALT (all *p*-values > 0.05).

**Table 3:** Partial correlations between ANGPTL8 levels and clinical variables.

	**Model 1**		Model 2^*^		Model 3^*^	
	**R**	***P*-value**	**Partial r**	***P*-value**	**Partial r**	***P*-value**
Age	0.24	0.000	0.19	0.002	0.20	0.001
BMI	0.02	0.710	0.06	0.323	0.15	0.022
eGFR	−0.26	0.000	−0.26	0.000	−0.27	0.000
Creatinine	0.28	0.000	0.29	0.000	0.30	0.000
HbA1c	0.03	0.621	−0.05	0.424	−0.06	0.381
HDL	−0.01	0.887	−0.02	0.711	−0.07	0.309
LDL	−0.09	0.154	−0.15	0.014	−0.13	0.037
Total cholesterol	−0.07	0.246	−0.10	0.099	0.09	0.159
TG	−0.03	0.684	0.05	0.397	−0.03	0.624
FPG	0.04	0.496	−0.04	0.477	−0.04	0.586
2h PG	0.12	0.048	0.01	0.901	0.09	0.167
ALT	0.04	0.534	0.08	0.223	−0.09	0.141
AST	0.10	0.091	0.24	0.000	0.25	0.000

* *When a variable was calculated in the Partial correlation, it would not be included in adjustment model*.

*Model 1 was unadjusted*.

*Model 2 was adjusted for age, sex and BMI*.

*Model 3 was adjusted for all variables in model 2 plus, HbA1c, HDL, LDL, TG, cholesterol, FPG, 2h PG, ALT, AST, DM, and hyperlipidaemia*.

### Subgroup Analyses

**Figure 2:**
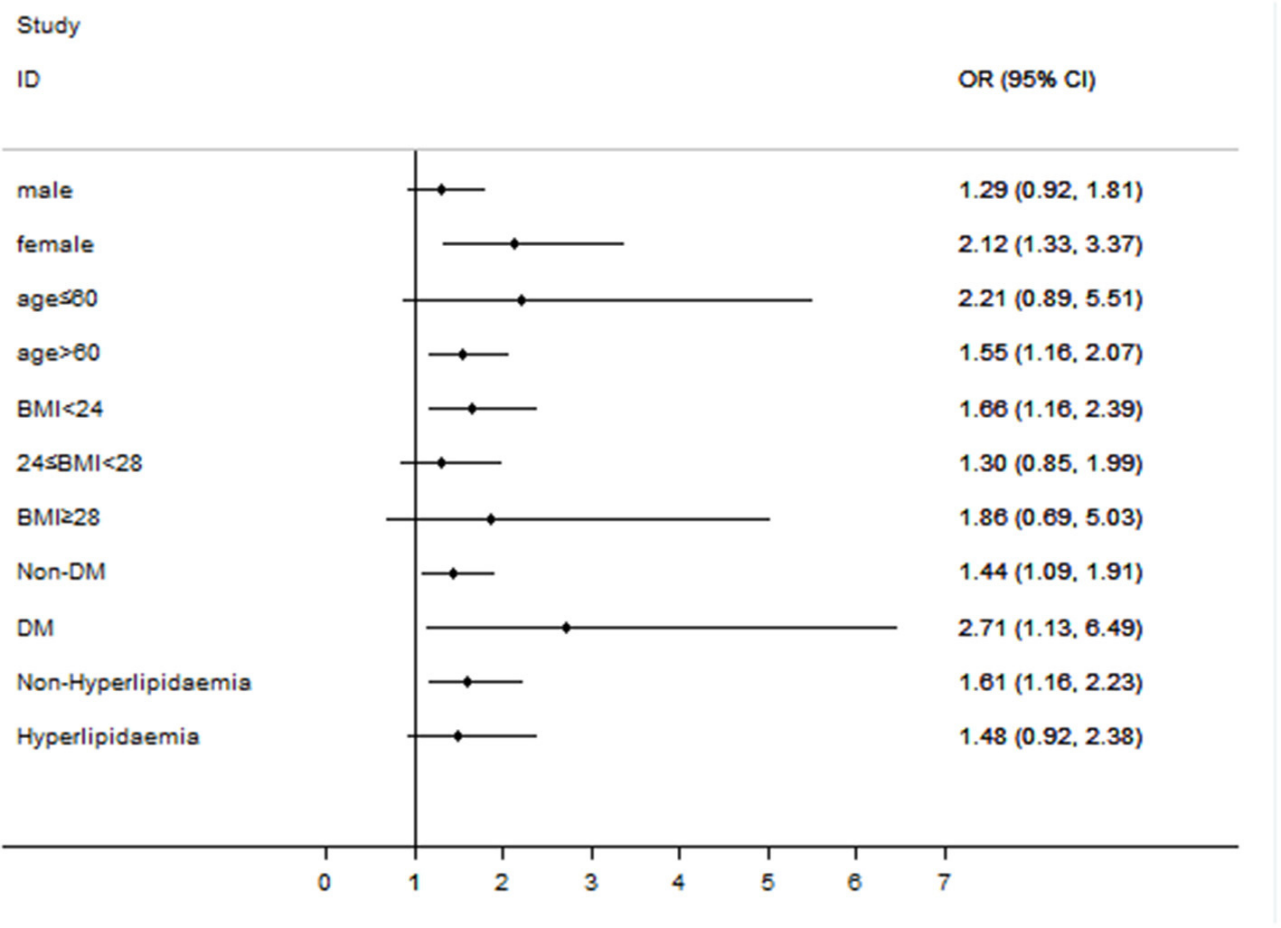
Subgroup analyses: the relationship between ANGPTL8 levels and renal dysfunction varied among subgroups divided according to sex, age, BMI, and underlying disease status. Subgroup analyses of the OR of RD according to a 1-SD increase in ANGPTL8 levels. The final model was adjusted for age, sex, BMI, diabetes, and hyperlipidaemia, except for the stratifying variable.

As shown in Figure 2, the association between ANGPTL8 levels and RD was consistent with the overall findings in both non-diabetic individuals (OR, 1.44; 95% CI, 1.09-1.91) and diabetic patients (OR, 2.71; 95% CI, 1.13-6.49). Furthermore, the estimates for this association were also significant in females (OR, 2.12; 95% CI, 1.33-3.37), individuals aged > 60 (OR, 1.55; 95% CI, 1.16-2.07), individuals with a BMI < 24 (OR, 1.66; 95% CI, 1.16-2.39), and individuals without hyperlipidaemia (OR, 1.61; 95% CI, 1.16-2.23) (all *p*-values <0.05). However, no significant association was found between ANGPTL8 levels and RD in male subjects, individuals aged ≤ 60, individuals with a BMI ≥ 24, or individuals with hyperlipidaemia (all *p*-values > 0.05).

## Discussion

In our study, we found that circulating ANGPTL8 levels were higher in patients with RD and negatively correlated with RD in the general population. Furthermore, multivariable-adjusted binary logistic regression analysis showed that patients in the highest quartile of ANGPTL8 levels had a 3.8-fold higher risk of RD than those in the lowest quartile. Moreover, subgroup analyses revealed significant differences among subgroups divided according to female sex, age > 60 years, BMI < 24, non-DM status, DM status, and non-hyperlipidaemia status.

The accumulating data on ANGPTL8 levels imply a complex role in metabolic function, potentially involving cross talk between lipid and glucose regulation (22). Previous studies found that circulating ANGPTL8 levels increased in diabetic patients (23). Furthermore, other studies demonstrated that circulating ANGPTL8 levels were much higher in patients with diabetic nephropathy than in diabetic patients without RD (14–16). Issa et al. proposed that ANGPTL8 could be used to predict the incidence of DM, and they also concluded that changes in renal function significantly decreased DM risk (24). Interestingly, the present study found that the association between ANGPTL8 levels and CKD existed not only in diabetic patients but also in non-diabetic individuals, indicating that ANGPTL8 may play a role in kidney disease, not just high glucose-mediated damage in diabetic nephropathy.

However, our case-control study could not identify the causal mechanisms driving the observed associations between ANGPTL8 levels and RD. A possible explanation for the involvement of increased circulating ANGPTL8 levels in impaired renal functions is that RD impairs the excretion of ANGPTL8. In this case, the elevated ANGPTL8 levels in individuals with RD may be a consequence of—not the cause of—CKD. However, this theory was challenged by the study of Chen et al. (25). They found that there were increased urinary ANGPTL8 levels in type 2 diabetic patients compared with healthy subjects, but there were no differences among the normoalbuminuria, microalbuminuria, and macroalbuminuria groups. This finding ruled out the possibility that reduced clearance of ANGPTL8 resulted from decreased glomerular filtration in the different groups.

Another possible mechanism is that the high serum levels of ANGPTL8 impair renal function, but the biological mechanism remains unclear. First, we found that ANGPTL8 levels were positively correlated with age, which was also reported in several previous studies (11, 15, 23, 26–28). Furthermore, subgroup analyses also suggested that the association between ANGPTL8 levels and RD was more significant in older participants. With increasing age, proinflammatory cytokine levels in the body increase (29, 30). Abu-Farha et al. also found that ANGPTL8 levels positively correlated with high sensitivity C-reactive protein (HsCRP) levels in humans (27). Moreover, chronic inflammation is a common feature in RD due to multiple factors, such as the induction of proinflammatory cytokines, oxidative stress, uraemia, and high rates of infection (2, 31). Therefore, age-related inflammation may mediate the association between ANGPTL8 levels and RD. Second, ANGPTL8 plays a critical role in lipoprotein and triglyceride regulation (16, 26, 28, 32, 33). Lipid accumulation in the renal parenchyma is detrimental to renal function (34). The study of Yang et al. suggested that triglycerides might partially mediate the correlation between ANGPTL8 levels and the ACR (17). Moreover, elevated plasma levels of ANGPTL8 are associated with atherosclerosis (28, 35). Recently, it has been appreciated that severe occlusive disease of the renal artery results in chronic renal ischaemia and can lead to the development of chronic renal insufficiency (36, 37). Therefore, ANGPTL8 may cause RD by damaging the renal artery.

### Limitations

Some limitations of this study need to be acknowledged. First, all of the participants in our study were Chinese, limiting the generalizability of the findings, which must be confirmed in other ethnic groups. Second, the cross-sectional study design used here limits our interpretation of the causal relationship driving the observed associations between ANGPTL8 levels and RD events. Further prospective cohort studies are needed to answer this question. Finally, the sample size of our study was relatively small, and further physiological studies are needed to determine the causal links and underlying mechanisms.

## Conclusion

In conclusion, increased circulating ANGPTL8 levels were associated with an increased risk of RD in the general population. Furthermore, subgroup analyses indicated that the relationship between ANGPTL8 levels and RD was significant in females, individuals aged > 60, individuals with a BMI < 24, individuals without DM, individuals with DM, and individuals without hyperlipidaemia. ANGPTL8, a newly discovered protein involved in a variety of pathological and physiological processes, may be closely related to CKD. This implies that ANGPTL8 may play a role in the pathological process of RD and provides a foundation for further exploration of the causes of chronic kidney dysfunction. Nevertheless, future longitudinal studies with longer follow-ups are warranted to explain the causal relationship between ANGPTL8 levels and RD; moreover, research should continue to explore the relevant mechanisms.

## Data Availability Statement

The datasets used and/or analyzed during the current study are available from the corresponding author on reasonable request.

## Ethics Statement

The studies involving human participants were reviewed and approved by Tongji Hospital, Tongji Medical College, Huazhong University of Science and Technology. The patients/participants provided their written informed consent to participate in this study.

## Author Contributions

XY and XM designed the study. XM, HZ, DL, PY, and LH collected the data. XM and HZ performed the serum ANGPTL8 measurements and the statistical analysis. XY and XM wrote the paper. DL, PY, LH, JZ, and WL reviewed the paper and provided suggestions. XY is the guarantor of this work, had full access to all the data in the study, and takes responsibility for the integrity of the data. All authors reviewed the manuscript.

## Funding

This study was supported by grants from the National Key R&D Program of China (2016YFC0901203) and the National Natural Science Foundation of China (81570740 and 81974109).

## Conflict of Interest

The authors declare that the research was conducted in the absence of any commercial or financial relationships that could be construed as a potential conflict of interest.

## Publisher's Note

All claims expressed in this article are solely those of the authors and do not necessarily represent those of their affiliated organizations, or those of the publisher, the editors and the reviewers. Any product that may be evaluated in this article, or claim that may be made by its manufacturer, is not guaranteed or endorsed by the publisher.
